# Retraction Note: MiR-200c/FUT4 axis prevents the proliferation of colon cancer cells by downregulating the Wnt/β-catenin pathway

**DOI:** 10.1186/s12885-023-11471-4

**Published:** 2023-10-09

**Authors:** Jinchun Cong, Jian Gong, Chuanjia Yang, Zhixiu Xia, Hong Zhang

**Affiliations:** 1 Department of General Surgery, Shengjing Hospital China Medical University, No. 36 Sanhao Street, Heping District, Shenyang, 110004 China; 2 Department of Clinical Pharmacy, Shenyang Pharmaceutical University, Shenyang, 110016 China

**Retraction Note: BMC Cancer (2020) 21:2**

10.1186/s12885-020-07670-y

The Editors have retracted this article due to the authors’ inability to provide documentation of approval from the ethics committee. The authors have not responded to correspondence from the publishers about this retraction.

